# High-Gain Photonic Crystal Antenna Based on Tamm Plasmon Polaritons

**DOI:** 10.3390/mi17080914

**Published:** 2026-07-29

**Authors:** Mingyang Liu, Guang Lu, Bing Wang, Hao Zhang

**Affiliations:** 1 School of Space Science and Technology, Shandong University at Weihai, Weihai 264209, China; 202538111@mail.sdu.edu.cn (M.L.); wbing@sdu.edu.cn (B.W.); 2 Laboratory for Electromagnetic Detection (LEAD), Institute of Space Sciences, Shandong University, Weihai 264209, China; 202437630@mail.sdu.edu.cn

**Keywords:** antenna, photonic crystal, Tamm plasmon polaritons, high gain

## Abstract

To overcome the large size of conventional high-gain antennas and the structural complexity of typical photonic crystal antennas, this paper proposes a cavity-free photonic crystal (PC) antenna driven by microwave-band Tamm plasmon polaritons (TPPs), which eliminates the conventional half-wavelength resonant cavity while maintaining a moderate total height (38.4 mm, ~2.1λ_0_). The core innovation of this work lies in shifting the gain-enhancement paradigm from traditional bulky, volume-based spatial resonances to a direct 2-D interface feeding strategy. By rigorously satisfying the phase-matching condition between a one-dimensional PC and a highly reflective substrate, a strong TPP mode is excited. Distinct from conventional designs, we embed a simple microstrip patch exactly at this phase-matched boundary to directly exploit the extreme electric field localization of TPPs. This novel mechanism enables a cavity-free architecture that achieves highly directional emission without complex feeding networks or metallic cavities. Simulations and measurements exhibit excellent agreement. At 16.43 GHz, the measured peak gain reaches 16.4 dBi, with 3-dB beamwidths of 13.5° and 18.5°. Ultimately, this TPP-driven paradigm offers a practical solution tailored for advanced wireless communications and radio astronomy.

## 1. Introduction

With the rapid development of modern wireless communication and radar systems, there is an increasingly urgent demand for compact antennas with high gain and high directivity [1,2]. Traditional gain-enhancement methods, such as utilizing large parabolic reflectors or complex antenna arrays, often suffer from inherent physical limitations, including large physical dimensions, high profiles, and feeding network losses [3,4]. To overcome these constraints, antennas based on photonic crystal (PC) structures and metasurfaces have attracted widespread attention in academia in recent years [5,6,7,8]. Recently, advanced metasurfaces and dielectric photonic crystals have emerged as versatile platforms for wave manipulation and beamforming, enabling highly directive leaky-wave radiation and multi-user spot-beamforming [X4–X7] [7,8,9,10]. Owing to their unique photonic bandgap (PBG) effects and photon localization characteristics [11,12], PCs have shown great potential in the design of low-profile, highly directional antennas. However, existing PC antennas typically rely on the vertical stacking of multilayer dielectrics to construct Fabry-Pérot resonant cavities [7,8,11], or require the introduction of defects into two- or three-dimensional periodic lattices to achieve electromagnetic mode localization [12,13]. This inherently introduces a fundamental trade-off between large physical dimensions and structural complexity. Therefore, exploring novel mechanisms to achieve highly directional emission without relying on complex feeding networks or bulky spatial reflecting cavities remains a critical challenge.

Tamm plasmon polaritons (TPPs) are localized boundary states trapped at the interface of two distinct highly reflective media [14,15], such as a metal and a Bragg reflector [16]. Surface plasmons and localized plasmonic resonances are also well known for their strong field confinement and have been widely used in applications such as high-performance SPR sensors and multi-band tunable absorbers [X1–X3] [17,18,19]. Unlike conventional surface plasmon polaritons (SPPs)—which are non-radiative surface waves typically requiring complex momentum-matching structures [20,21]—TPPs can be directly excited by TE and TM waves at arbitrary incident angles without additional structures [22,23]. Furthermore, distinct from traditional Fabry-Pérot (F-P) cavities, which rely on bulky volume-based spatial resonances [24,25,26,27], TPPs achieve intense field localization directly at a two-dimensional physical interface. Despite their successful application in optical devices (e.g., absorbers [28,29], lasers [30], and sensors [31]), extending TPPs to the microwave band remains challenging. Moreover, extending Tamm resonances to 1-D microwave photonic crystal systems with conductive boundaries has recently opened up new possibilities for interfacial electromagnetic mode tailoring [32]. Consequently, utilizing TPPs to circumvent the radiation bottlenecks of traditional antennas represents a compelling research frontier [32,33].

To address these challenges, this paper introduces TPPs into the microwave band to design a compact, high-gain antenna. By combining a 1-D PC with a reflective substrate, we satisfy the phase-matching condition to excite a strong TPP mode. Unlike bulky resonance-based designs, this approach embeds a microstrip patch at the interface, exploiting intense TPP energy concentration for radiation. Furthermore, bypassing strict quarter-wavelength constraints [34,35], this mechanism achieves extreme field localization via interfacial phase matching. Simulation and measurement results demonstrate high gain and low sidelobe performance, eliminating complex feeding networks and bulky cavities. Ultimately, this work offers a practical approach for miniaturized microwave antennas.

## 2. Antenna Design and Operating Principle

**Figure 1:**
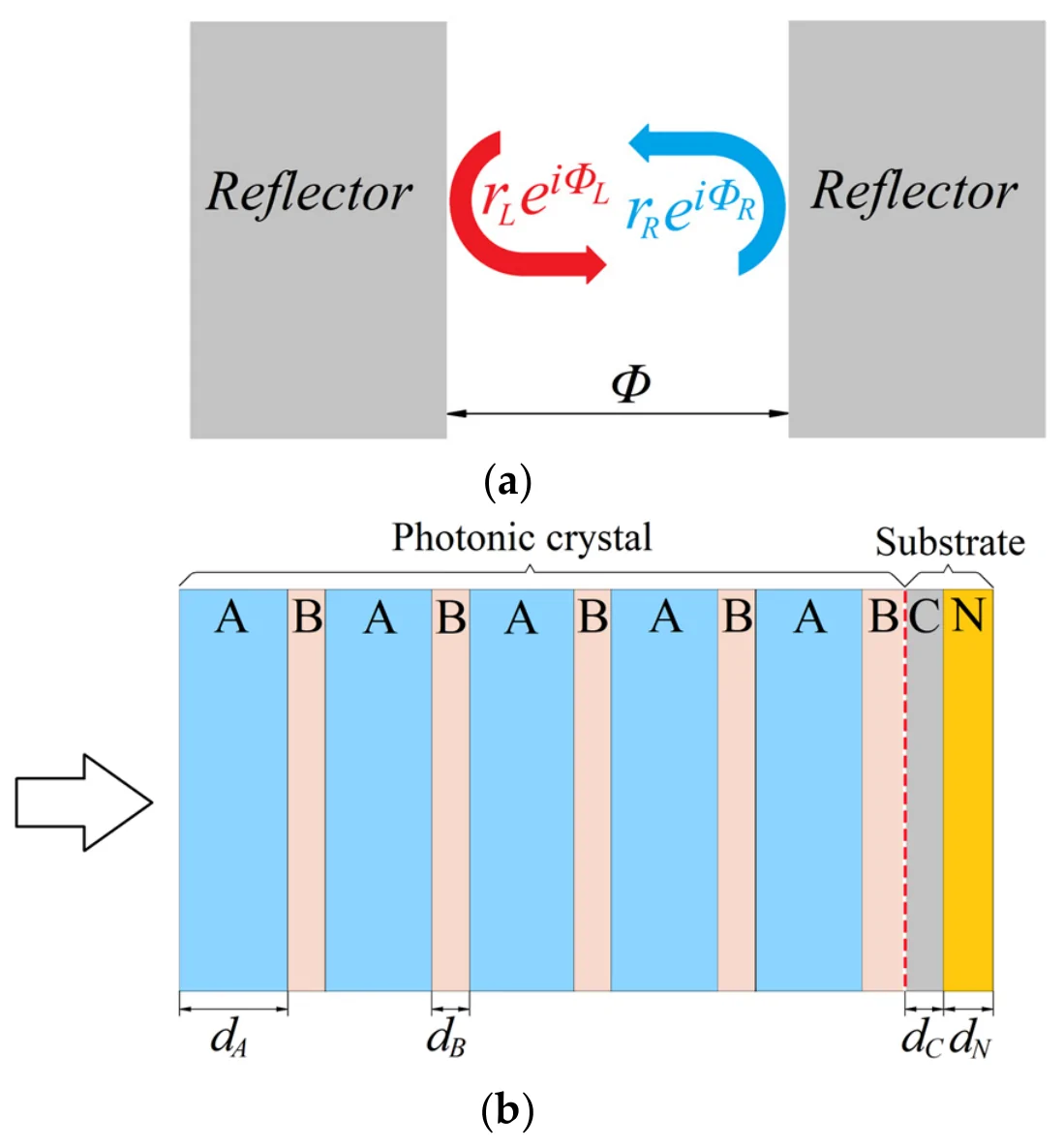
(**a**) Schematic of the TPPs excitation mechanism in a microcavity with complex reflection coefficients. (**b**) Schematic of the (AB)^5^CN composite structure for TPPs excitation.

To excite Tamm plasmon polaritons (TPPs) at the interface of a composite structure composed of two highly reflective materials, as shown in Figure 1a, a rigorous phase-matching condition is strictly required [14,15]. When electromagnetic waves impinge on the structure, the physical boundary separating these two distinct media functions as an effective virtual microcavity, as illustrated in Figure 1a. $r_{L}e^{-i\Phi_{L}}$ and $r_{R}e^{-i\Phi_{R}}$ denote the complex reflection coefficients of the left and right materials, respectively. The formation of the TPPs is fundamentally governed by the constructive interference of multiple reflected waves at this interface, which leads to a dramatic resonant enhancement of the localized electromagnetic field. Accounting for a propagation phase Φ induced by the discontinuity of the refractive index within the cavity, the general resonance condition is expressed as:

(1) $${r_{L}r}_{R}e^{-i{(\Phi }_{L}+\Phi_{R})}e^{-i\left(2\Phi \right)}=1$$

When the two reflective structures are in direct physical contact, the equivalent cavity length diminishes to zero, yielding no propagation phase delay. Consequently, the resonance equation simplifies to:

(2) $${r_{L}r}_{R}e^{-i{(\Phi }_{L}+\Phi_{R})}=1$$

Given that the constituent materials are highly reflective, the amplitude condition is inherently fulfilled. Therefore, the excitation of the interface state is strictly dictated by the simplified phase-matching criterion [14,28]:

(3) $$\Phi_{L}+\Phi_{R}=0$$

Based on the phase condition (3) for exciting TPPs, we designed a multilayer structure denoted as (AB)^5^CN, as shown in Figure 1b. This structure comprises a periodic photonic crystal (AB)^5^ and a highly reflective substrate CN, designed to excite TPPs at their interface by leveraging their respective reflection characteristics. The photonic crystal is formed by periodically alternating layers of dielectric plate A (permittivity *ε*_A_ = 1, thickness *d*_A_ = 6 mm) and plate B (permittivity *ε*_B_ = 3.38, thickness *d*_B_ = 1.524 mm), with a period number of 5. The highly reflective substrate consists of a dielectric plate C (permittivity *ε*_C_ = 3.38, thickness *d*_C_ = 0.813 mm) and a copper layer *N*. The physical thicknesses of the dielectric plate and the air gap were primarily selected based on the commercial availability of the Rogers RO4003C substrate and the ease of structural assembly.

**Figure 2:**
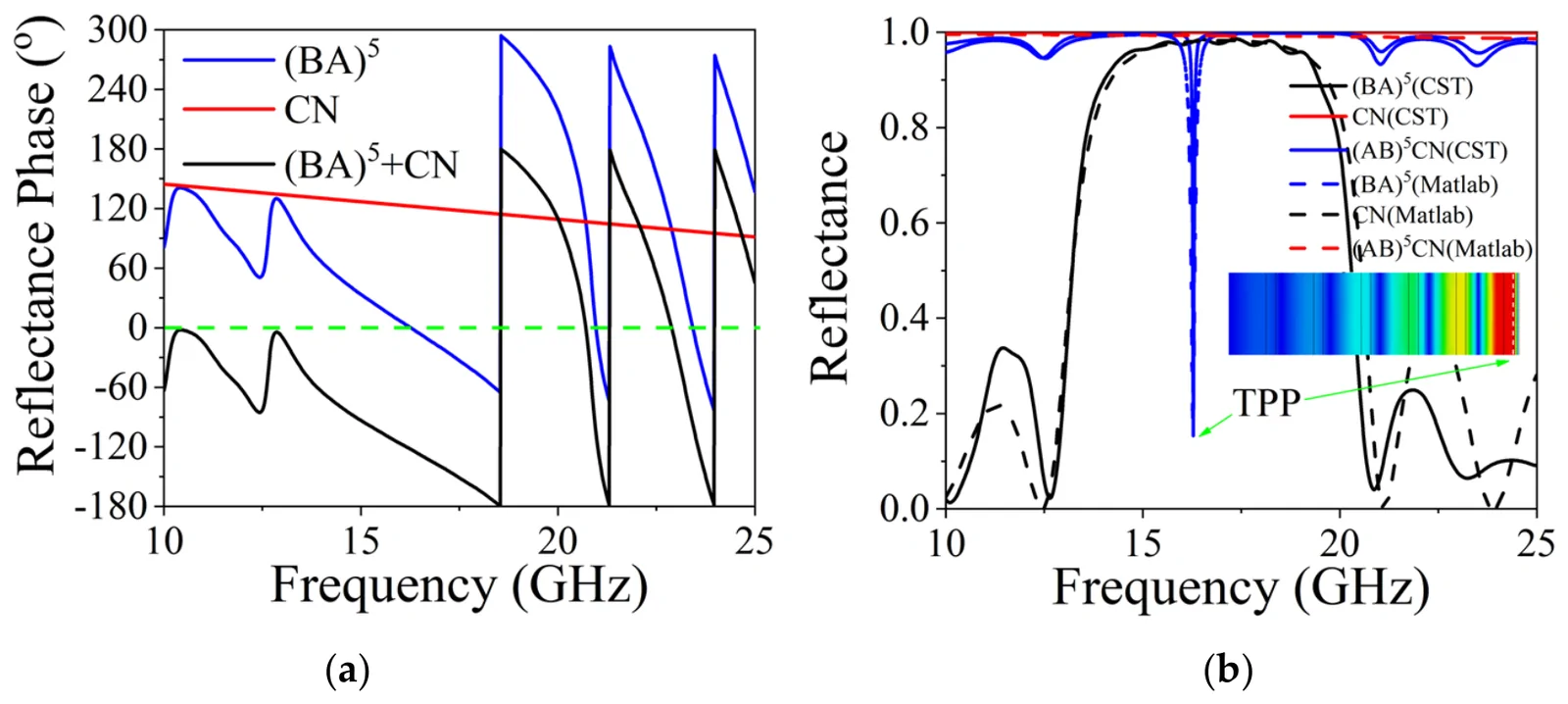
(**a**) Simulated reflection phase spectra of (BA)^5^, CN, and their superposition. (**b**) Simulated reflection spectra of (BA)^5^, CN, and (AB)^5^CN.

To verify the feasibility of this structure, the reflection phase spectra of the photonic crystal (AB)^5^ and the highly reflective substrate CN were calculated independently using CST Microwave Studio. Figure 2a presents the calculated reflection phase spectra for the (BA)5 and CN structures, as well as the direct superposition of their reflection phases. The phase superposition curve clearly shows that at 16.28 GHz, the sum of their reflection phases is exactly zero, satisfying the excitation condition for TPPs. Furthermore, the electromagnetic response of the composite structure was analyzed. Figure 2b plots the calculated reflection spectra for the (BA)^5^, CN, and (AB)^5^CN structures. The results indicate that the (AB)^5^CN structure successfully excites the TPPs at 16.28 GHz, manifesting as an extremely sharp reflection dip. Combined with the normalized electric field distribution shown in the inset of Figure 2b, it is evident that the electromagnetic energy is strongly localized at the interface between the (AB)^5^ and CN layers. This localization achieves a significant electric field enhancement, physically verifying the successful excitation of the Tamm state from the perspective of field distribution. To verify the CST simulations, the reflection spectra were analytically calculated using the transfer matrix method (TMM) in MATLAB [14,36], showing excellent agreement between both methods.

To analytically verify the reflection spectrum, the transfer method (TMM) is utilized. For a single dielectric layer *i*, the propagation matrix M*_i_*, which relates tangential electric and magnetic fields at the input and output interfaces of that layer, is expressed as:

(4) $$M_{i}=\left(\begin{array}{cc} \cos\delta_{i} & -\frac{i}{\eta_{i}}\sin\delta_{i}\\ -i\eta_{i}\sin\delta_{i} & \cos\delta_{i} \end{array}\right)$$

where $\delta_{i}=k_{z,i}d_{i}$ is the phase shift and $\eta_{i}$ is the wave admittance of layer *i*. The overall transfer matrix of the *N*-layer composite structure is obtained via chain multiplication:

(5) $$M_{\mathrm{total}}=\prod_{i=1}^{N}M_{i}=\left(\begin{array}{cc} M_{11} & M_{12}\\ M_{21} & M_{22} \end{array}\right)$$

The complex reflection coefficient *r* is then directly derived as:

(6) $$r=\frac{\left(M_{11}+M_{12}\eta_{0})\eta_{0}-(M_{21}+M_{22}\eta_{0}\right)}{\left(M_{11}+M_{12}\eta_{0})\eta_{0}+(M_{21}+M_{22}\eta_{0}\right)}$$

Finally, the reflectance is given by $R={\left|r\right|}^{2}$.

**Figure 3:**
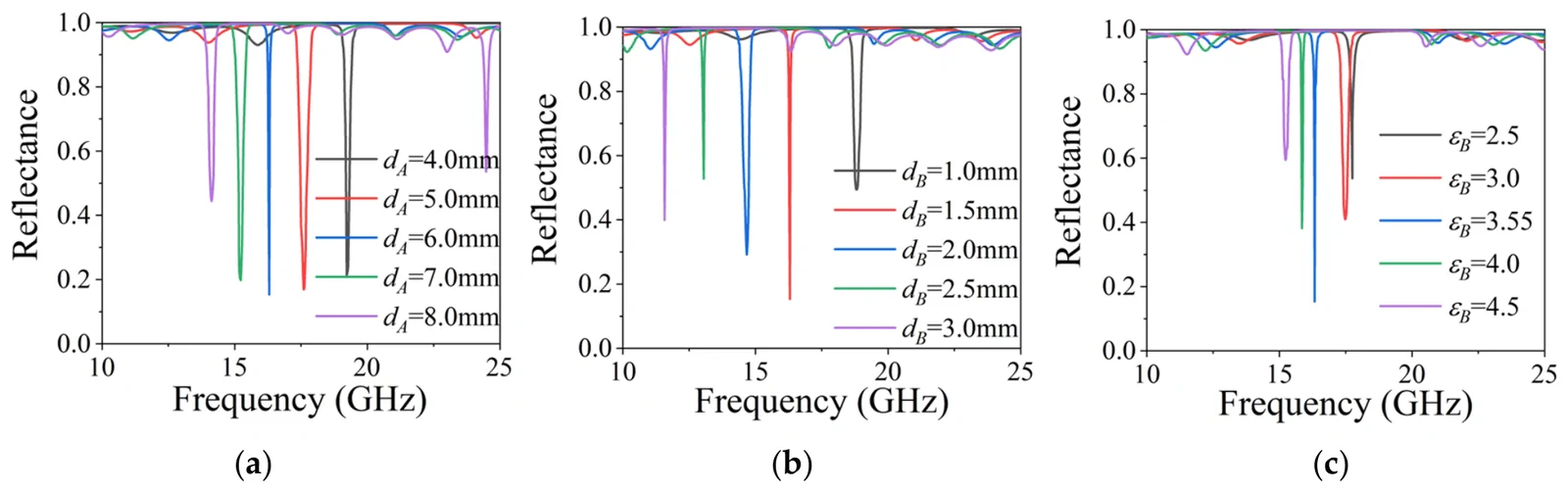
Simulated reflection spectra of the (AB)^5^CN structure with varying (**a**) thickness *d*_A_, (**b**) thickness *d*_B_, and (**c**) permittivity *ε*_B_.

Illustrates the dependence of the TPP resonance on structural parameters. Increasing the layer thickness (*d*_A_, *d*_B_) or permittivity (*ε*_B_) consistently red-shifts the excitation frequency, demonstrating that the operating band can be flexibly scaled by tuning these parameters, as shown in Figure 3. As the air gap thickness *d*_A_ increases from 4.0 mm to 8.0 mm, the TPP resonant frequency red-shifts from 19.3 GHz to 14.2 GHz, corresponding to a total frequency shift of Δ*f* = 5.1 GHz. As the dielectric thickness *d*_B_ increases from 1.0 mm to 3.0 mm, the resonance shifts from 18.8 GHz to 11.6 GHz (Δ*f* = 7.2 GHz), indicating a higher frequency sensitivity to dB. As the relative permittivity *ε*_B_ increases from 2.5 to 4.5, the resonant frequency shifts from 17.8 GHz to 15.2 GHz (Δ*f* = 2.6 GHz). These quantitative metrics explicitly confirm that the operating band can be flexibly scaled across a broad frequency range by finely tuning the physical dimensions and material parameters.

**Figure 4:**
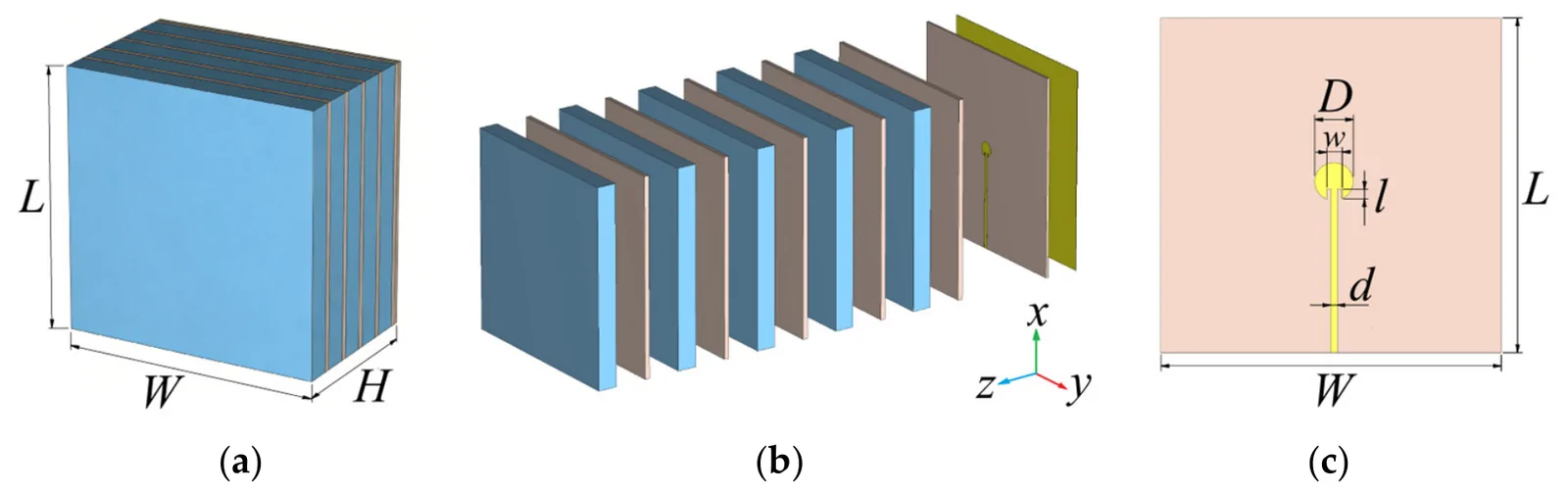
(**a**) Overall 3D schematic of the proposed antenna. (**b**) Exploded view of the layered structure. (**c**) Layout of the radiating patch layer.

Enabled by the field localization effect of TPPs, electromagnetic signals are locally enhanced at the interface. Exploiting this physical mechanism, a high-performance, high-gain antenna can be constructed by placing a microstrip patch—acting as a signal radiator or receiver—directly at the interface where the electric field enhancement occurs. Figure 4 details the configuration of the proposed antenna, including (a) a schematic of the overall structure, (b) an exploded view of the layered structure, and (c) the layout of the radiating patch layer. The overall dimensions of the antenna are length *L* = 60.0 mm, width *W* = 60.0 mm, and height *H* = 38.4 mm. For the radiating patch, the circular patch diameter is *D* = 4.6 mm, the feed line width is *d* = 1.2 mm, and the slot dimensions are *w* = 2.0 mm and *l* = 1.3 mm.

## 3. Simulation and Measurement Results

**Figure 5:**
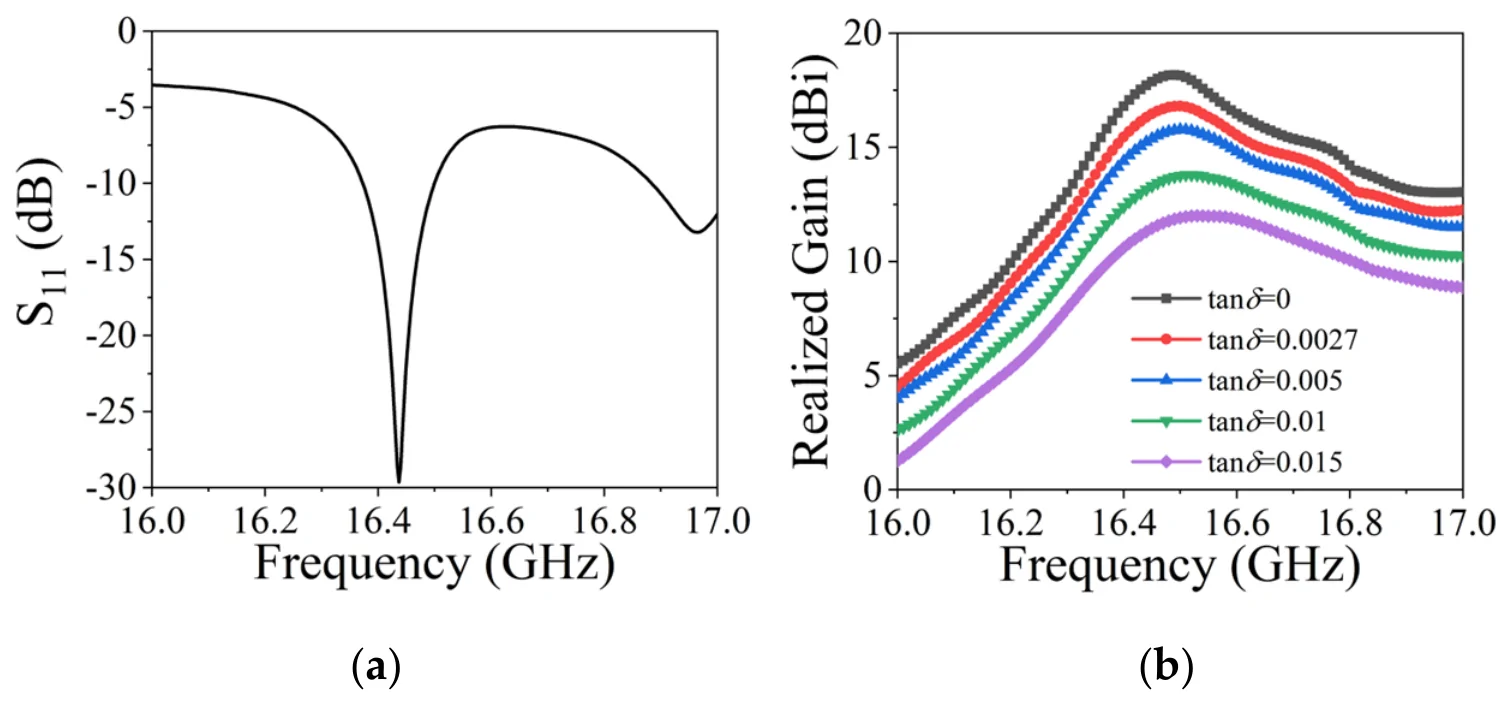
(**a**) Simulated S_11_ and (**b**) realized gain curve of the antenna.

Full-wave electromagnetic simulations were carried out using CST Microwave Studio to assess the performance of the proposed antenna. Figure 5a depicts the simulated S_11_ response. The results show that the antenna maintains an S_11_ < −10 dB over the frequency range of 16.37–16.50 GHz, demonstrating good impedance matching. Within this operating band, the antenna demonstrates excellent high-gain radiation performance. As illustrated in Figure 5b, the dielectric loss tangent (tan*δ*) significantly impacts the radiation performance; a consistent degradation in the overall gain is observed as the material loss increases. For the selected substrate with tan*δ* = 0.0027, the realized gain remains above 15.94 dBi throughout the operating bandwidth, achieving a peak gain of 16.7 dBi at 16.42 GHz. Notably, the antenna’s resonant frequency slightly deviates from the TPPs excitation frequency. This shift is primarily attributed to edge effects, which arise from the discrepancy between the infinite multilayer structure assumed in theoretical calculations and the finite dimensions of the physical antenna.

**Figure 6:**
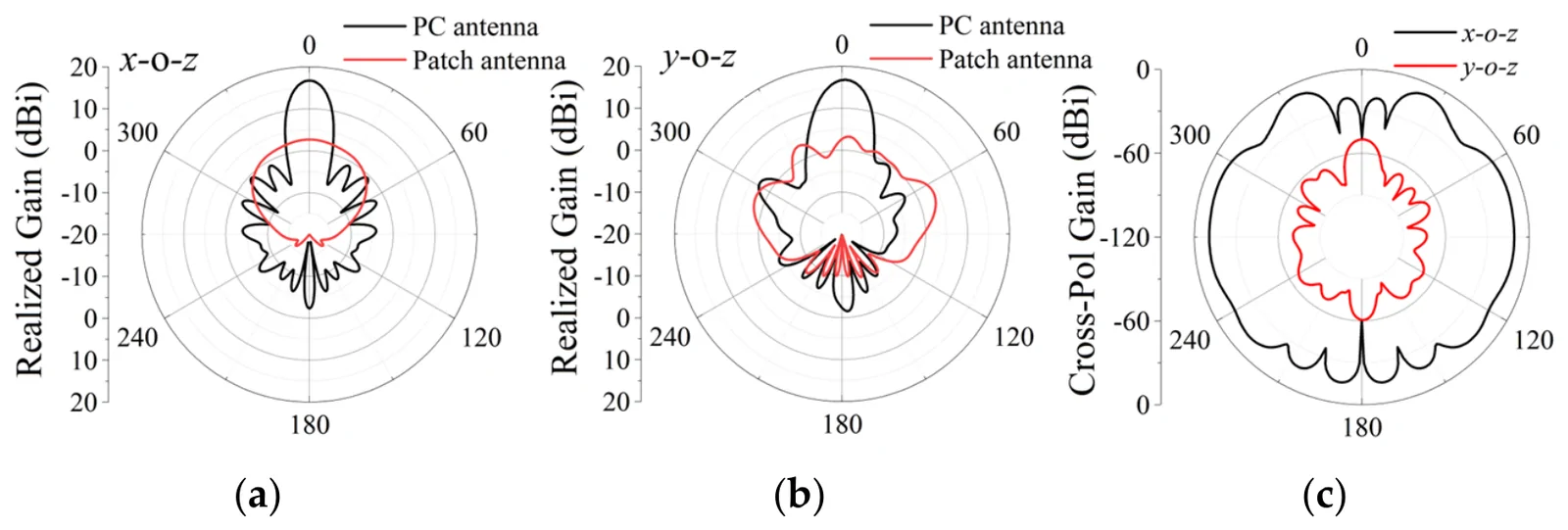
(**a**) Simulated radiation patterns (x-o-z plane) of the PC antenna and patch antenna at 16.42 GHz. (**b**) Simulated radiation patterns (y-o-z plane) of the PC antenna and patch antenna at 16.42 GHz. (**c**) Simulated cross-polarization radiation patterns of the PC antenna at 16.42 GHz.

The black curves in Figure 6a,b plot the simulated radiation patterns of the proposed antenna at 16.42 GHz in the x-o-z and y-o-z planes, respectively. In the x-o-z plane, the antenna has a 3-dB beamwidth of 14° and a peak gain of 16.7 dBi, with the sidelobe level effectively suppressed to −17.4 dB. In the y-o-z plane, the 3-dB beamwidth is 17.4°, the gain is 16.7 dBi, and the sidelobe level is −13.1 dB. The simulated aperture efficiency is 34.5%. The red curves in Figure 6a,b represent the patterns of a bare radiating patch. Compared to this bare patch, the directivity is significantly enhanced, and the peak realized gain increases remarkably from 2.65 dBi to 16.7 dBi, achieving a substantial gain enhancement of 14.05 dB. These simulation results verify that by utilizing the Tamm state interface mode, the designed antenna successfully achieves the expected high gain and highly directional performance. As shown in Figure 6c, the proposed antenna exhibits excellent cross-polarization performance.

**Figure 7:**
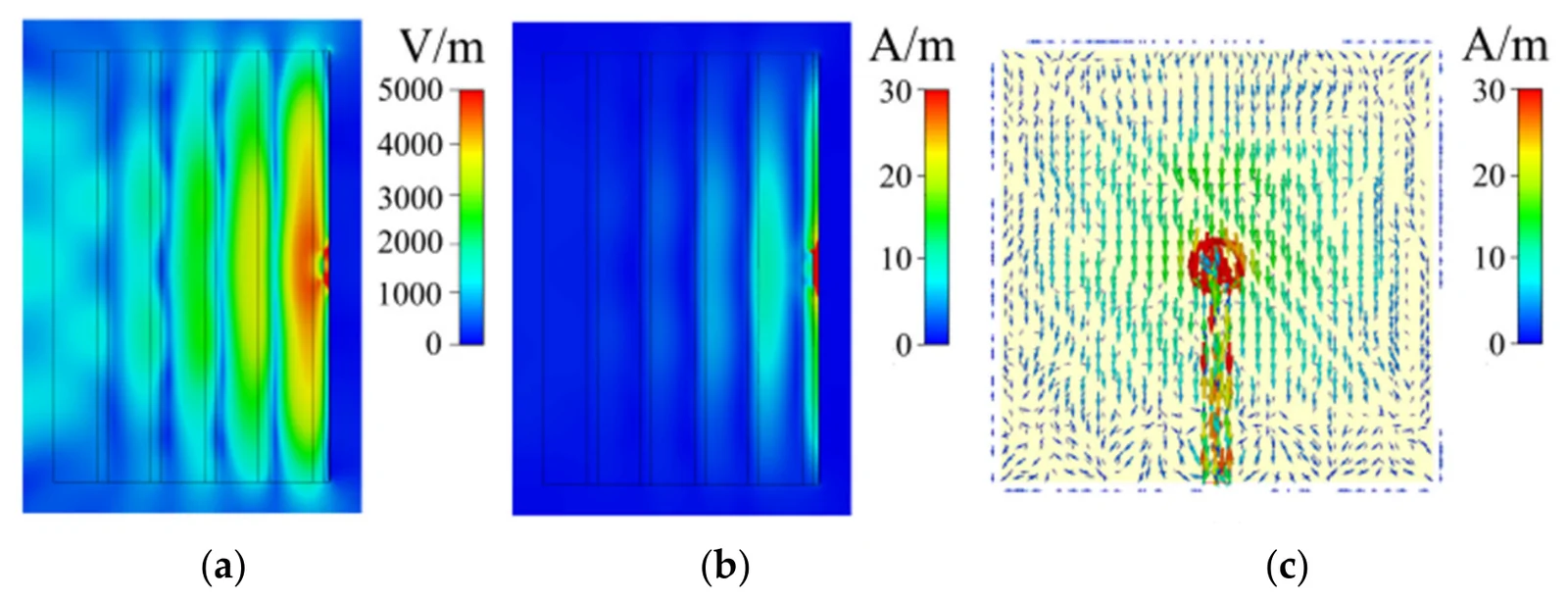
Simulated near-field distributions of the proposed antenna at 16.42 GHz: (**a**) electric field magnitude, (**b**) magnetic field magnitude and (**c**) surface current distribution.

As shown in Figure 7a,b, the electric and magnetic fields are tightly bound directly at the 2D contact boundary between the PC and the substrate, decaying exponentially into both media away from the interface. This distinct spatial pattern fundamentally differs from traditional Fabry-Pérot (F-P) resonant cavities, which inherently rely on spatial standing waves distributed across a voluminous half-wavelength dielectric or air cavity. By completely eliminating the bulky spatial cavity, our TPP architecture achieves extreme interfacial electric field concentration (>5000 V/m) at a cavity-free interface. Furthermore, the strong surface current localized on the embedded microstrip patch (Figure 7c) directly confirms efficient energy coupling from this interface-confined mode to directional broadside radiation, demonstrating the physical essence of our volume-free resonance enhancement.

It should be noted that the overall height of the proposed structure is 38.4 mm (~2.1λ_0_ at 16.43 GHz). This height is not a resonant cavity but is primarily determined by the five-period Bragg mirror (AB)^5^, which is required to achieve the necessary reflection phase for TPP excitation. The TPP resonance itself is localized at the interface and does not rely on a spatial standing-wave cavity; the field decays exponentially away from the interface, as will be shown in the near-field distributions. Therefore, the antenna is ‘cavity-free’ in the sense of eliminating the dedicated half-wavelength resonant volume, despite having a finite physical thickness.

**Figure 8:**
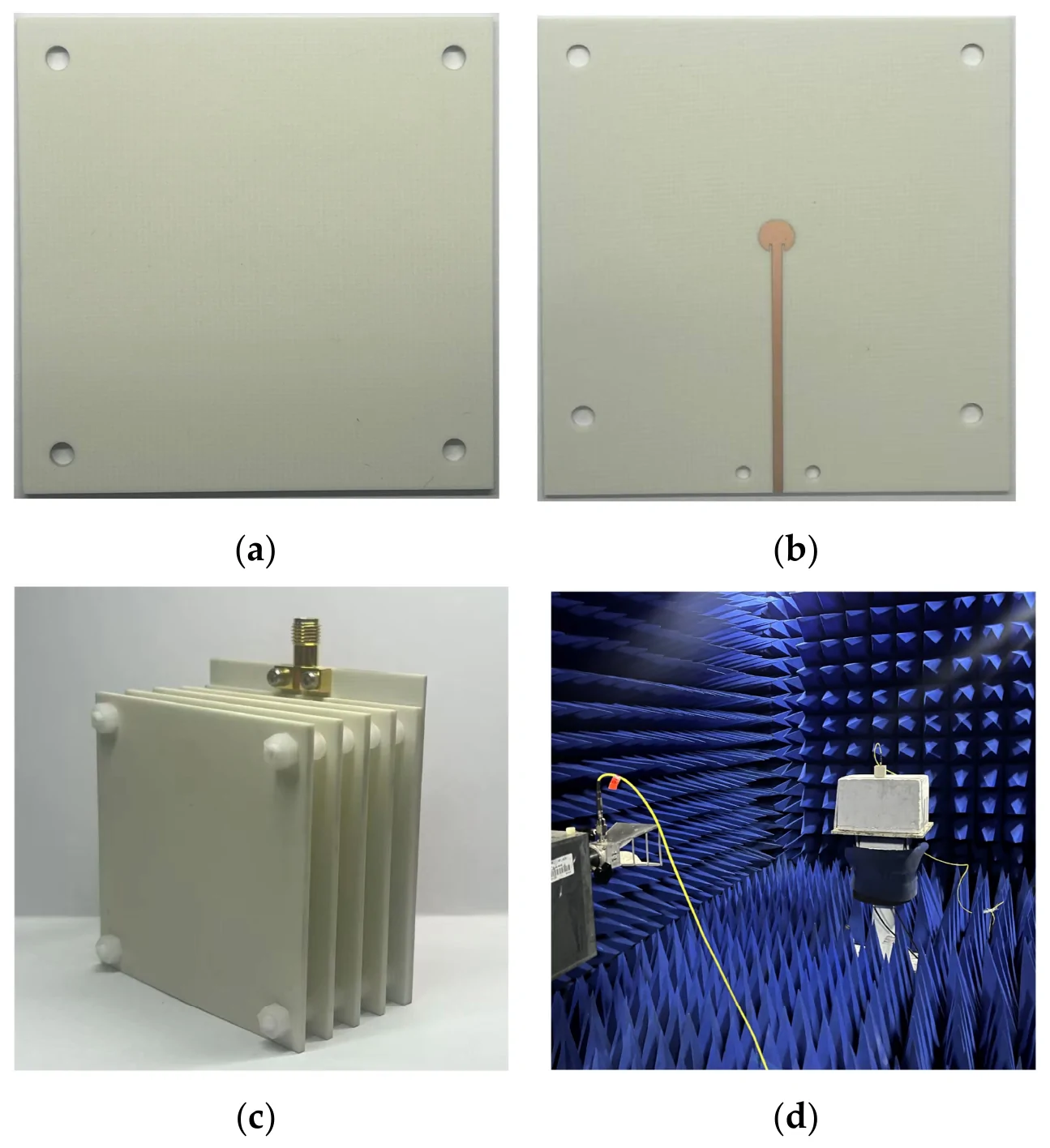
(**a**) Schematic of photonic crystal layers. (**b**) Schematic of the CN substrate. (**c**) Photograph of the fabricated antenna. (**d**) Measurement scene of the antenna in a microwave anechoic chamber.

To verify the accuracy of the theoretical design and simulation results, a prototype of the antenna was fabricated using standard printed circuit board (PCB) technology. As shown in Figure 8a, the high-refractive-index layers of the photonic crystal employ 1.524-mm-thick Rogers RO4003C substrates (relative permittivity *ε* = 3.38, tan*δ* = 0.0027), while the low-refractive-index layers are 6-mm-thick air gaps. The highly reflective layer utilizes a 0.813-mm-thick Rogers RO4003C board, with solid copper cladding on the bottom side and the radiating metal patch on the top side, as shown in Figure 8b. A microstrip line with an SMA connector is used for feeding. Figure 8c shows a photograph of the fabricated antenna. The S_11_ parameter was measured using a calibrated Keysight N5225B PNA network analyzer. Furthermore, the far-field radiation performance was evaluated in a standard microwave anechoic chamber. Figure 8d displays the antenna prototype placed inside the chamber during the measurement.

**Figure 9:**
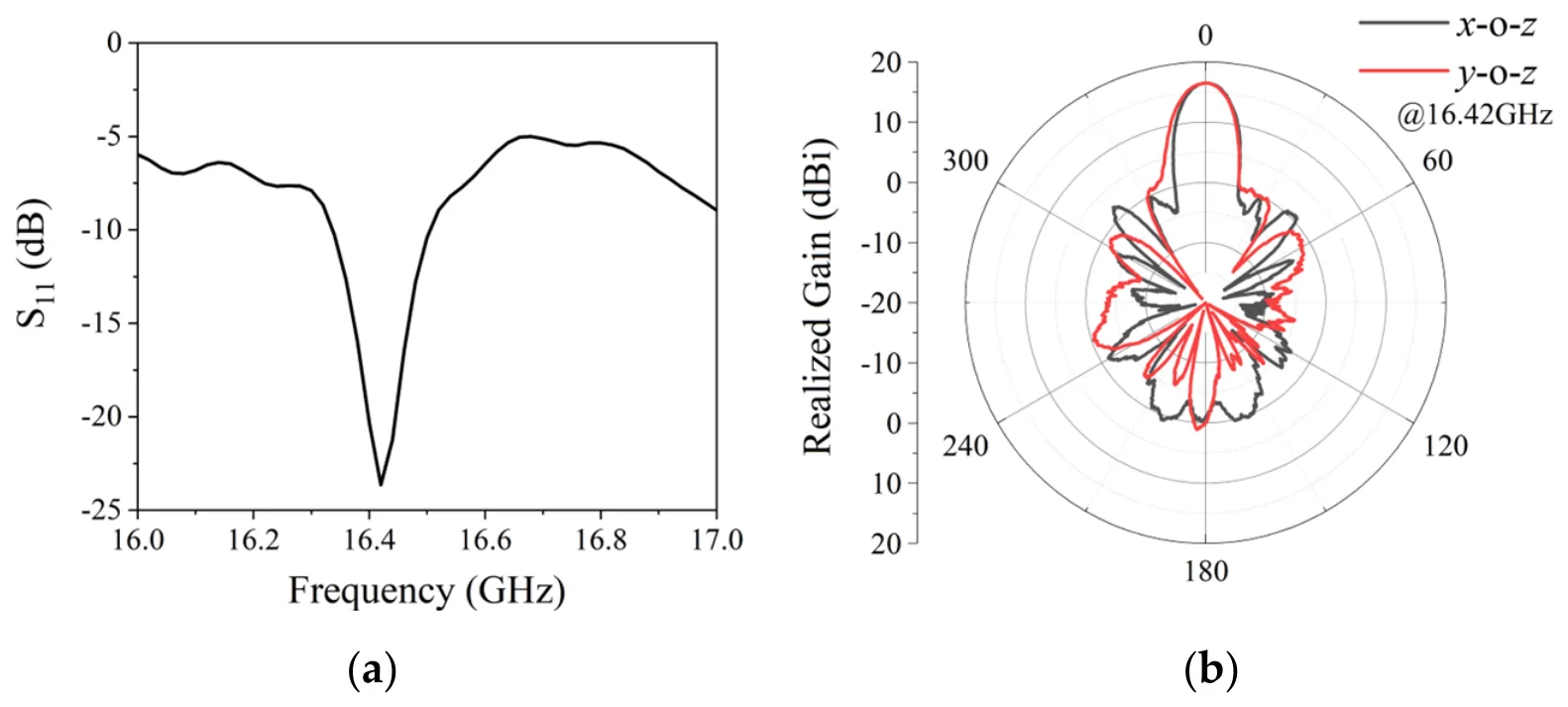
Measured (**a**) S_11_ and (**b**) radiation patterns.

Figure 9a shows the measured results for the S_11_ parameter and radiation patterns. The measured S11 indicates that the effective operating band (S_11_ < −10 dB) covers the range from 16.34 GHz to 16.50 GHz. The experimental results agree well with the simulations. The slight frequency shift is primarily attributed to fabrication tolerances and minor fluctuations in the substrate’s permittivity. Regarding the far-field radiation characteristics, the measured pattern at 16.43 GHz in the x-o-z plane shows a 3-dB beamwidth of 13.5°, a sidelobe level of −16.6 dB, and a realized gain of 16.4 dBi. In the y-o-z plane, the measured 3-dB beamwidth is 18.5°, the sidelobe level is −15.4 dB, and the realized gain is 16.4 dBi. The measured aperture efficiency is 32.2%. Table 1 summarizes the comparison between the measured and simulated results. This excellent agreement proves that the microwave photonic crystal antenna based on optical TPPs successfully achieves high gain and highly directional radiation.

Compared with other referenced designs, the proposed antenna requires no complex superstrate but a simple 1-D periodic dielectric plate. This scheme completely eliminates physical resonant cavities yet maintains high gain and aperture efficiency. Comparison with other jobs is shown in Table 2. Additionally, while the high-Q characteristic leads to a narrow bandwidth, it inherently provides the antenna with excellent filtering performance. Overall, this design successfully achieves a balance between a simple structure, a low profile, and highly directional radiation. To broaden the bandwidth in future designs, feasible approaches include cascading photonic crystals for multi-mode TPPs coupling or employing 2-D/3-D structures for multi-dimensional dispersion engineering.

## 4. Conclusions

In summary, a compact, high-gain photonic crystal antenna based on Tamm plasmon polariton (TPP) excitation is proposed and experimentally validated. The core innovation of this work lies in shifting the gain-enhancement paradigm from traditional volume-based spatial resonances to a direct 2-D interface feeding strategy. By embedding a simple patch precisely at the phase-matched boundary, the antenna directly leverages the extreme electric field localization of TPPs. This mechanism enables a cavity-free interface—completely eliminating complex feeding networks and metallic reflectors—while achieving an excellent measured peak gain of 16.4 dBi at 16.43 GHz.

Compared with other high-gain antennas, this antenna has no resonant cavity and uses an easy-to-make 1D photonic crystal structure. Conventional Fabry-Pérot antennas rely on thick resonant dielectric layers, and antennas based on surface plasmon polaritons can only reach low gain values. In comparison, the TPP interface resonance breaks the trade-off between antenna height and radiation performance. However, the antenna only provides a 3.7% working bandwidth. This limitation comes from the high-Q feature of TPP modes and restricts its use in wideband communication equipment. Small frequency deviations between simulation and measurement results are caused by machining errors of dielectric thickness and air gap size. To overcome the narrow bandwidth inherent to the high-Q single-mode TPP excitation, two practical structural enhancement strategies can be implemented in future iterations: The impedance bandwidth can be significantly broadened to 10% by either grading the photonic crystal periods to trigger multi-mode TPP coupling or integrating a multi-resonant metasurface patch to form a hybrid dual-resonance system. Moreover, the TPP excitation method proposed in this paper can be applied to various planar antennas, which helps develop more small-sized high-gain antennas for radio astronomy and millimeter-wave communication systems.

## Figures and Tables

**Table 1:** Comparison of Simulated and Measured Performances.

	Frequency (GHz)	Realized Gain (dBi)	Angular Width (^o^)	Side Lobe Level (dB)
Simulated	16.42	16.7	x-o-z: 14.0; y-o-z: 17.4	x-o-z: 17.4; y-o-z: 13.1
Measured	16.43	16.4	x-o-z: 13.5; y-o-z: 18.5	x-o-z: 16.6; y-o-z: 15.4

**Table 2:** Performance Comparison with Existing Superstrate-Based High-Gain Antennas.

References	Superstrate	Center Frequency (GHz)/Bandwidth	Size (λ_0_ × λ_0_)	Cavity Size	Gain (dBi)	Aperture Efficiency
[11]	2D PC	24/narrow	4.3 × 1.6	λ_0_/2	14	29.3%
[24]	MTM	14.2/18.7%	5.8 × 5.8	λ_0_/2	13.78	22.6%
[25]	AMC	2.4/39.6%	1.4 × 1.4	λ_0_/6	12.5	58.3%
[26]	FSS	8.35/3%	3.5 × 3.5	λ_0_/2	19.7	60%
[27]	PRS	7.6/24.7%	2.2 × 2.2	λ_0_/2	13.2	36.2%
[21]	SPP	15.0/59.76%	1.8 × 1.65	no	10.6	30.76%
This work	1D PC	16.43/3.7%	3.2 × 3.2	zero	16.4	32.2%

## Data Availability

The data that support the plots within this paper and other findings of this study are available from the corresponding authors on reasonable request.
